# 2D in-Plane Ordered MXene Nanosheets Derived from (Mo_2/3_Er_1/3_)_2_AlC Rare-Earth i‑MAX for Energy Storage Applications

**DOI:** 10.1021/acsanm.5c04789

**Published:** 2026-01-06

**Authors:** Nisha Hiralal Makani, Chandra M. Adhikari, Shanna Marie M. Alonzo, Bishnu Prasad Bastakoti, Binod K. Rai, Bhoj Raj Gautam

**Affiliations:** † Department of Chemistry, Physics, and Materials Science, 3338 Fayetteville State University, Fayetteville, North Carolina 28301, United States; ‡ Department of Chemistry, North Carolina A&T State University, Greensboro, North Carolina 27411, United States; § 1073 Savannah River National Laboratory, Aiken, South Carolina 29808, United States

**Keywords:** i-MAX, i-MXenes, rare earth, vacancy, structural, electrochemical, electronic

## Abstract

MXenes have become one of the most versatile families of two-dimensional (2D) materials due to their high conductivity, hydrophilicity, and remarkable electrochemical performance. This has stimulated intense efforts to design and synthesize MXenes, including structurally unique in-plane ordered 2D MXenes called i-MXenes. Here, we have synthesized the quaternary rare earth (RE)-based i-MAX phase (Mo_2/3_Er_1/3_)_2_AlC using an arc melting method, and the corresponding 2D i-MXene was then obtained through a LiF/HCl soft etching process. Literature studies have shown that Al and the RE element are etched out during the etching process, leading to the formation of pure vacancy-ordered Mo_1.33_C 2D i-MXene. However, our investigation reveals that upon exposure to a fluorine solution, the i-MAX phase forms RE fluoride impurities, which are challenging to remove through HCl–DI water washing and persist in the final product, resulting in impure Mo_1.33_C@Er i-MXene. These results were confirmed by various characterizations such as X-ray diffraction, Raman spectroscopy, X-ray photoelectron spectroscopy, and scanning transmission electron microscopy. Although the Mo_1.33_C@Er electrode showed a 24-fold increase in specific capacitance compared to its parent i-MAX phase, it still exhibited a high charge-transfer resistance arising from the insulating nature of RE fluoride byproducts, which adversely influence the overall capacitance behavior of the synthesized 2D Mo_1.33_C@Er i-MXenes. This study contributes to identifying pathways for the preparation of pure 2D i-MXenes from RE-based i-MAX phases and developing improved synthesis methods. With additional process optimization, the 2D i-MXene holds a strong potential for electrochemical energy storage applications. Additionally, the electronic structures of Mo_1.33_C were theoretically studied using first-principles density functional theory calculations, which revealed that pristine Mo_1.33_C is metallic, and this metallic nature is preserved even with –O, –F, and mixed functionalization.

## Graphical abstract

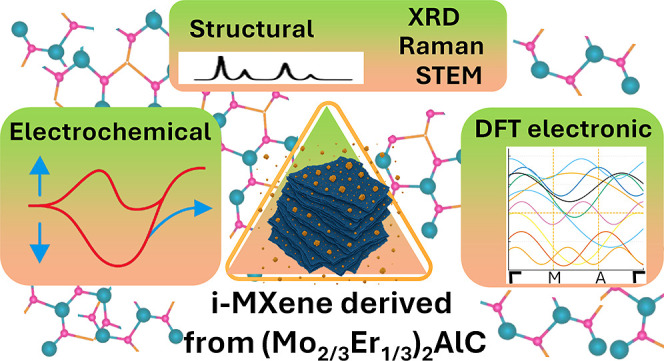

## 1 Introduction

In 2024, the International Union of Pure and Applied Chemistry recognized two-dimensional (2D) MXenes in its Top Ten Emerging Technologies in Chemistry, underscoring their significance in advancing chemical sciences,^1^ which highlights that MXenes are widely researched materials with a broad range of applications, particularly in areas such as energy storage field due to their exceptional conductivity and chemical stability.^2–6^ MXenes are 2D transition-metal carbides or nitrides produced by etching the A element from MAX phases.^3,7,8^ MAX phases are layered ternary phases with the general formula M_*n*+1_AX_*n*_ (where M is an early transition metal, A is an element from groups 13–14, and X is carbon and/or nitrogen).^9,10^ The etching process, commonly carried out using fluoride-containing solutions such as HF or LiF/HCl, removes the A layer while preserving the M–X slabs, yielding 2D sheets with surface terminations (T*x* = –F, –O, –OH).^7,11,12^ Therefore, the general formula for MXenes is given by M_n+1_X_n_T_*x*_. The first 2D MXene, Ti_3_C_2_T_*x*_, was reported in 2011 and synthesized from the Ti_3_AlC_2_ MAX phase by etching the Al.^13^ Subsequently, several MAX phases with single transition metals, such as Nb_2_AlC, V_2_AlC, and Ti_3_Al­(CN), were developed, along with their corresponding MXenes.^14–18^ In 2015, MAX phases featuring double transition metals with ordered structures were reported, such as Mo_2_Ti_2_AlC_3_ and Cr_2_TiAlC_2_.^19^ These structures consist of one or two metal layers sandwiched between two transition metal layers and were termed out-of-plane ordered (o-MAX) phases, with their corresponding MXenes named o-MXenes.^20^ Over the years, research on MAX phases has rapidly progressed in the pursuit of novel MXenes. In 2017, a new family of in-plane ordered MAX phases, known as i-MAX phases, was introduced.^21^ These quaternary MAX phases follow the general formula (M_2/3_′M_1/3_″)_2_AX, where M′ and M″ can include elements such as Sc, Y, Zr, Hf, V, Cr, Mo, Mn, and W in a 2:1 ratio.^22–31^ The A element is either Al or Ga, while X is C. Additionally, i-MAX phases were synthesized utilizing rare-earth (RE) elements, including Gd, Tb, Dy, Ho, Er, Y, Tm, Yb, and Lu.^24,32–36^ In the i-MAX phase, M′ atoms form a honeycomb lattice, while M″ atoms fill the centers of the hexagons. The extrusion of M″ atoms from the M′ layers toward the A layer results in a Kagome lattice in either a monoclinic lattice structure (space group *C*2/*c*, *C*2*/m*) or an orthorhombic lattice structure (space group *Cmcm*).^21,33,37^

The first i-MAX phase synthesized was (Mo_2/3_Sc_1/3_)_2_AlC, and from this, the first 2D i-MXene was derived.^21^ This process led to the discovery of novel vacancy-ordered Mo_1.33_C 2D i-MXenes, as both Al and Sc were etched during the etching process. Subsequent electrochemical measurements revealed exceptionally low resistivity and high capacitance values.^21^ Moreover, asymmetric supercapacitors constructed from 2D i-MXene (Mo_1.33_C//Mn_*x*_O_*n*_) exhibit outstanding cycling stability;^38^ building on these studies, new 2D i-MXenes W_1.33_C were developed.^39–41^ Meshkian et al.^41^ presented a combined theoretical and experimental study on the structural investigation of vacancy-ordered 2D i-MXene, demonstrating that W_1.33_C synthesized from two different parent materials (such as (W_2/3_Sc_1/3_)_2_AlC and (W_2/3_Y_1/3_)_2_AlC) exhibits distinct surface functional groups, which are known to significantly influence their fundamental properties.^41^ Persson et al.^42^ studied the selective etching of the M″ and A elements, demonstrating that by adjusting the etchant concentration, selective etching can be achieved, leading to different compositions of 2D i-MXenes.^42^ Additionally, 2D i-MXenes Mo_1.33_C and W_1.33_C were synthesized from rare earth (RE)-based i-MAX phases, and their electrochemical performance was thoroughly examined.^43,44^ The capacitance of Mo_1.33_C, derived from (Mo_2/3_RE_1/3_)_2_AlC, varied depending on the specific RE element used.^44^ Furthermore, Chen et al.^45^ synthesized (W_2/3_Mo_2/3_)C 2D i-MXene from the solid solution (W_1/3_Mo_1/3_RE_1/3_)_2_AlC i-MAX phase, and a specific capacitance of 120 F/g at 1 A/g was obtained. It was further treated with hydrazine monohydrate (N_2_H_4_·H_2_O) and rGO, which resulted in an increase in specific capacitance due to the presence of more O-/N-containing surface terminations and the formation of an interactive microstructure that improved the overall conductivity of the electrode.^45,46^ In addition to experimental investigations, several theoretical studies have also been carried out on 2D i-MXenes. Mostafaei et al.^47,48^ examined the electronic structure and optical properties of ((Mo/W)_2/3_(Sc/Y)_1/3_)_2_CO_2_ 2D i-MXenes, and Khazaei et al.^49^ focused on the electronic structure of functionalized 2D (Mo_2/3_Y_1/3_)_2_C. These works revealed that O-functionalized i-MXenes exhibit semiconducting behavior, whereas F-functionalized counterparts are metallic in nature.^50^

As mentioned in the above studies, during the synthesis of 2D i-MXenes from the quaternary i-MAX phase, M″ (Sc, Y, and RE) elements can also be etched out by reacting with fluoride ions, forming byproducts, similar to the reaction between A elements and fluorides.^21,43,44^ In conventional MAX phases, the A elements, usually aluminum, react with fluoride to form AlF_3_, which easily dissolves in HCl and deionized (DI) water, facilitating its removal. This process can be likened to separating a mixture of rock and salt, where the “rock” represents the MXene and the “salt” (the impurities or A- and M″-based fluoride byproduct) dissolves in solution and is eliminated by centrifugation. Existing literature on 2D i-MXenes generally reports that washing with HCl and DI water effectively removes A- and M″-element fluoride byproducts, resulting in a pure 2D i-MXene structure.^43,44^ Here, we employed a (Mo_2/3_Er_1/3_)_2_AlC RE-based i-MAX phase for the synthesis of vacancy-ordered Mo_1.33_C@Er using the conventional LiF/HCl etching method and carried out detailed structural characterization using X-ray diffraction (XRD), Raman spectroscopy, X-ray photoelectron spectroscopy (XPS), and scanning transmission electron Microscopy (STEM) of the resulting material. However, analyses present that prepared Mo_1.33_C@Er is not in a pure form and show some impurity presence of Er-based fluoride byproducts, indicating that the standard synthesis and/or washing procedure may be insufficient. To assess the effect of byproducts on electrochemical behavior, comprehensive capacitance studies were conducted through cyclic voltammetry (CV), galvanostatic charge-discharge (GCD), and electrochemical impedance spectroscopy (EIS). Additionally, first-principles calculations have been performed on Mo_1.33_C 2D i-MXenes with various functional groups and their combinations, providing insights into the material’s electronic nature.

## 2 Experimental Procedure

### 2.1 Synthesis of the (Mo_2/3_Er_1/3_)_2_AlC i-MAX Phase

To prepare the i-MAX phase (Mo_2/3_Er_1/3_)_2_AlC polycrystalline samples, a nominal composition of all required materials was used in a conventional arc melting setup under an argon atmosphere with a water-cooled copper hearth. The materials used included Mo (Thermo Scientific, powder, 99.95%), C (Sigma-Aldrich, powder, 99.99%), Er (Ames Laboratory), and Al (Alfa Aesar, 99.999%). Initially, Mo and C powders were mixed in the appropriate atomic weight ratio (Mo:C = 1.333:1) using a pestle and mortar, and a homogeneous powder was formed, which was then mechanically pressed. Subsequently, Er and Al pieces were added to the pressed powder in an appropriate weight ratio (Mo_1.33_C/Er/Al = 1:0.666:1) composition, and the mixture was arc-melted using an Edmund Buhler electric arc furnace.^31^ The alloy was flipped and remelted 4–5 times to ensure a homogeneous ingot.

### 2.2 Synthesis of 2D Mo_1.33_C@Er i-MXene

The conventional LiF/HCl method was used for synthesizing Mo_1.33_C@Er. Initially, 20 mL of 9 M HCl was added to a small Nalgene bottle, which was immersed in an oil bath to maintain a constant temperature of 70 °C. Next, 0.6 g of the (Mo_2/3_Er_1/3_)_2_AlC i-MAX phase and 0.4 g of LiF powder were gradually added to the solution. The mixture was stirred and allowed to etch for 7 days. After etching, the solution was transferred to a Falcon tube and washed once with HCl, and the supernatant was discarded. The sediment was then washed multiple times with DI water with a centrifugation speed of 4500 rpm for 20 min until the pH reached 6. Finally, the sediment was dried at 70 °C for 24 h to remove any residual solvent. A schematic presentation of the synthesis of 2D i-MXene is given in Figure 1a. To obtain delaminated nanosheets for characterization, 0.2 g of Mo_1.33_C@Er powder was mixed with 5 mL of TBAOH and vortexed for 20 min. The mixture was then centrifuged at 4500 rpm for 1 h to remove excess TBAOH. The sediment was washed with DI water and centrifuged again under the same conditions until the pH reached neutral. Subsequently, 5 mL of DI water was added, and a final low-speed centrifugation at 3500 rpm for 1 h was performed. The resulting bluish supernatant contained the delaminated nanosheets, which were used for HRTEM analysis.

**Figure 1:**
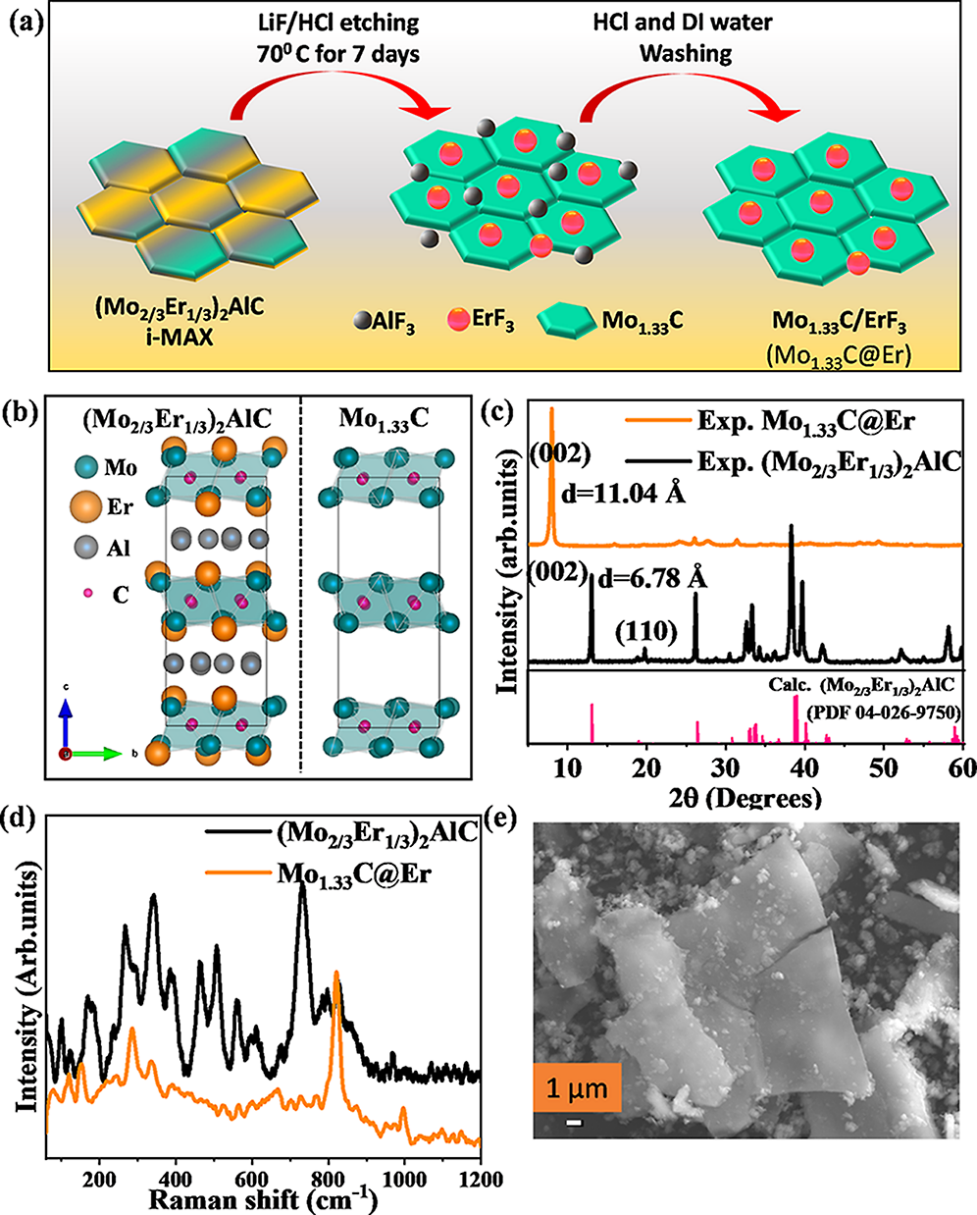
(a) Pictorial schematic depicting the synthesis of Mo_1.33_@Er, (b) structural representation, (c) XRD pattern, (d) Raman spectra of the (Mo_2/3_Er_1/3_)_2_AlC i-MAX phase and Mo_1.33_C@Er, and (e) SEM image of prepared Mo_1.33_C@Er.

## 3 Materials Characterization

The structural and morphological characterization of (Mo_2/3_Er_1/3_)_2_AlC and Mo_1.33_C@Er was carried out by using various techniques. XRD analysis was performed using a Rigaku diffractometer with a Cu Kα source (40 kV, 15 mA) over a 2θ range from 5 to 60°. Raman measurements were conducted using a 785 nm laser. For morphological analysis, scanning electron microscopy (SEM) was utilized. Atomic structure analysis experiments were performed on an Analytical Scanning Transmission Electron Microscope (STEM) Thermo Fisher Talos F200X.

## 4 Electrochemical Testing

Electrochemical measurements were carried out using a Biologic VMP3 workstation, with a Ag/AgCl electrode as the reference and a platinum electrode as the counter electrode. The working electrode slurry was prepared by mixing 5 mg of Mo_1.33_C@Er, 1 mg of poly­(vinylidene fluoride), and 100 μL of 1-methyl-2-pyrrolidinone. The mixture was sonicated for 1 h and subsequently brushed onto a microporous carbon cloth substrate (1.5 cm × 1.5 cm). The coated electrode was dried in an oven at 60 °C for 14 h.

## 5 Computational Method

First-principles density functional theory (DFT) calculations were performed using the Vienna Ab initio Simulation Package (VASP).^51,52^ Adapting initial structures from the experimentally observed (Mo_2/3_Er_1/3_)_2_AlC i-MAX phase with *C*2/*c* symmetry (space group number 15), crystal structures for 2D i-MXene and vacancy-containing Mo_1.33_C were created. In Mo_1.33_C i-MXene, Mo and vacancy (V) have in-plane chemical ordering in the metal layers, in which 2/3 of metal sites are Mo and 1/3 are V, such that the i-MXene sheet has a repeating three-site motif of (Mo–V–Mo). Vs occupy a distorted honeycomb sublattice in the a–c plane. Each V in Mo_1.33_C is surrounded by Mo atoms in all directions in such a way that V will not have another V as the nearest neighbor. The terminations (T_*x*_s) protrude, sitting above and below the surface metal atoms, forming Mo_1.33_CT_*x*_. Then, the crystal structures of bare and functionalized Mo_1.33_C were optimized, updating ionic positions, minimizing the crystals’ energy and forces, and treating electrons fully quantum mechanically. Projector augmented wave method-based pseudopotentials and Perdew–Burke–Ernzerhof functionals were used to address ion–electron interactions and exchange correlations, respectively.^53–55^ Energy and force convergence criteria of 10^–6^ eV and 10^–2^ eV/Å, respectively, and a plane wave cutoff energy of 520 eV were employed to fully relax the atomic coordinates. The Monkhorst–Pack Γ-centered *k*-point meshes were chosen for Brillouin zone sampling in such a way that the *k*-point spacing is approximately 0.2/Å in each direction. Electronic iterations were carried out using the blocked Davidson algorithm^56^ and reciprocal space projection operators. To avoid numerical problems due to the finite sampling of the Brillouin zone, Gaussian smearing with a width of 0.05 eV was employed. For each of the structures considered here, we have used a supercell of size 4 × 4 × 1.

## 6 Results and Discussion

### 6.1 Characterization of (Mo_2/3_Er_1/3_)_2_AlC i-MAX and Mo_1.33_C@Er i-MXene

Figure 1b illustrates the crystal structures of the i-MAX phase (Mo_2/3_Er_1/3_)_2_AlC and the corresponding i-MXene Mo_1.33_C, modeled by using VESTA software. The i-MAX phase exhibits a monoclinic structure with in-plane ordering of M′ and M″ atoms, which generates a (110) plane and shows a peak near 20° in the recorded XRD pattern, which is a fingerprint of the i-MAX phase, which is absent in traditional MAX phases.^22^ Here, the in-plane ordering peak for (Mo_2/3_Er_1/3_)_2_AlC i-MAX phase is at 19.89°, which matches well with the PCD database (1959003, PDF 04026-9750), confirming the formation of the i-MAX phase structure.^44^ The (002) diffraction peak shifts from 13.03° to 7.99°, indicating an expansion of the c-lattice parameter from 6.78 Å in the i-MAX phase to 11.04 Å in the multilayered solid-solution i-MXene after etching. The intensity of the (002) peak in Mo_1.33_C@Er is significantly higher compared with its i-MAX phase, suggesting a highly crystalline phase. Additionally, the XRD pattern of the etched material reveals new peaks at 24.6° and 27.8° and other minor peaks at higher degrees, absent in the pristine (Mo_2/3_Er_1/3_)_2_AlC phase. Matching these peaks with the ICDD database indicates the formation of ErF_3_,^57^ which is attributed to the reaction between Er and fluorine during etching, as also shown in Figure 1a. Another significant observation is the disappearance of the peak near 19.89° in the XRD pattern of Mo_1.33_C@Er, suggesting that the ordered arrangement of Mo and Er atoms in the i-MAX phase is disrupted following the removal of Er during etching. Once the ErF_3_ byproduct forms, unlike AlF_3_, it becomes insoluble in HCl and DI water, remaining in the prepared Mo_1.33_C@Er. Removing ErF_3_ is possible only using perchloric acid, a strong acid that may oxidize the resulting Mo_1.33_C@Er. This observation is further supported by XPS analysis and STEM measurements. The SEM micrograph (Figure S1) of i-MAX shows the irregular shape of the particles with sharp edges and varying thickness. Figure 1c shows the Raman spectra for (Mo_2/3_Er_1/3_)_2_AlC and its corresponding Mo_1.33_C@Er. The Raman shift wavenumbers are provided in Table S1. According to the study by Champagne et al.,^36,58^ theoretical phonon calculations show that Raman modes below ∼170 cm^–1^ arise from vibrations involving RE, Mo, and Al, ∼170–260 cm^–1^ modes are primarily associated with Mo and Al, and bands above ∼500 cm^–1^ correspond to C atom vibrations. We observed that closely spaced peaks were present in the (Mo_2/3_Er_1/3_)_2_AlC phase but absent in the Raman spectra of Mo_1.33_C@Er. Table S1 lists the possible vibrational modes for both samples, including contributions from erbium oxide and molybdenum oxide impurities present in the i-MAX phase,^44^ as well as residual ErF_3_ detected in Mo_1.33_C@Er, which also contributes to the observed Raman signals. The morphology of Mo_1.33_C@Er, as depicted in Figures 1e and S2a–d, transitions into a sheet-like morphology, a characteristic structure of 2D MXenes. Along with the sheets, small particulates are also observed, which likely correspond to residual ErF_3_ byproducts formed during etching.

XPS measurements were performed for (Mo_2/3_Er_1/3_)_2_AlC (Figures 2a–d and S3a–d) and Mo_1.33_C@Er (Figures 2e–h and S3e–h). Peak fitting and deconvolution were carried out for all elements, and the results were analyzed. The Mo 3d spectra (Figure 2a,e) exhibit Mo 3d_5/2_ and Mo 3d_3/2_ peaks for both samples. The Er 4d spectra (Figure 2b,f) display an Er_2_O_3_ peak in the i-MAX phase, indicating oxide-based impurities in pristine i-MAX. The presence of Er_2_O_3_ slightly affects the LiF/HCl etching process by consuming H^+^ and F^–^ and forming ErF_3_, which ultimately results in an impure Mo_1.33_C@Er. Additionally, the Er 4d spectra in Mo_1.33_C@Er show a shift to higher binding energy and the presence of an ErF_3_ (171.6 eV) peak, consistent with XRD results, suggesting that Er reacts with fluorine during the etching process and forms ErF_3_. The lower-energy peak at 170.1 eV is related to Er–O bonding, while the higher-energy peak corresponds to Er 4d_3/2_.^59^ The Al 2p peak (Figure 2c,g) is present in (Mo_2/3_Er_1/3_)_2_AlC but absent in Mo_1.33_C@Er, confirming the successful removal of Al. The C 1s spectra (Figure 2d,h) for the i-MAX phase show C–C and C–O peaks, and the 2D i-MXene displays a metal carbide, C–C, and carboxyl peak, further confirming 2D i-MXene formation. Figure S3a,e presents survey spectra, highlighting all the elements present in both samples. Specifically, the O 1s spectra (Figure S3b,f) for (Mo_2/3_Er_1/3_)_2_AlC indicate the presence of metal carbonates and oxides, while the O 1s spectra for Mo_1.33_C@Er show MoO_2_ and metal hydroxide peaks. Additionally, the Mo_1.33_C@Er spectra display the ErF_3_ peak in the F 1s spectra (Figure S3g) along with a Cl 2p peak (Figure S3h), both of which are absent in the (Mo_2/3_Er_1/3_)_2_AlC phase (Figure S3c,d), as chlorine and fluorine originate from the etchant. Accordingly, the termination stoichiometry of Mo_1.33_C@Er can be expressed as Mo_1.33_C O_0.46_(OH)_0.44_F_1.14_. These results confirm that the as-etched Mo_1.33_C@Er surface is predominantly fluorine-terminated with comparable but smaller contributions from –O and –OH terminations.

**Figure 2:**
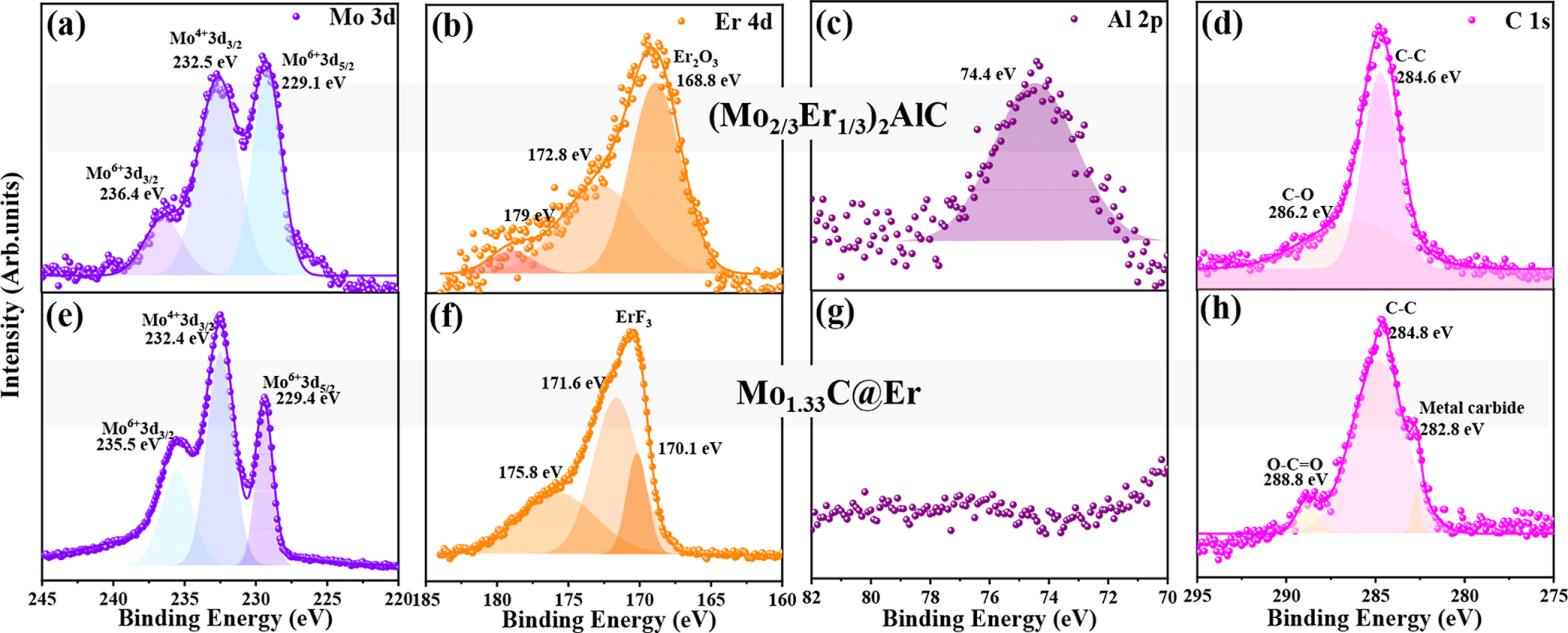
(a, e) Mo 3d, (b, f) Er 4d, (c, g) Al 2p, and (d, h) C 1s spectra of (Mo_2/3_Er_1/3_)_2_AlC and Mo_1.33_C@Er, respectively.

To investigate the structure of prepared Mo_1.33_C@Er, HRSTEM measurements were conducted using high-resolution Z contrast imaging with a high-angle dark field (HAADF) detector, as shown in Figure 3. For the analysis, a colloidal solution of Mo_1.33_C@Er was prepared using the TBAOH intercalation technique, resulting in a bluish solution (Figure S4). Figure S5 presents the HAADF image and elemental mapping of the (Mo_2/3_Er_1/3_)_2_AlC i-MAX phase (before etching). In the parent i-MAX structure, Mo and Er exhibit a similar distribution pattern. However, after etching, the elemental mapping of Mo and Er reveals a distinct change in their distribution. Figure 3a displays a lower magnification STEM image of Mo_1.33_C@Er. Higher magnification STEM images are shown in Figure 3b, b1,b2,b3 are enlarged views of the area of interest. As we observed in Figure 3b, the brightest spots in the image correspond to Er atoms, as Er is heavier than Mo and carbon; these bright dots are prominent in certain regions, highlighted with circles, while in other areas, they are less distinct, which suggest Er atoms are not uniformly distributed and instead form ErF_3_, which just remained in Mo_1.33_C@Er. Figure 3b1–b3, showing interplanar spacings of 0.32 and 0.17 nm, corresponding to the interplanar spacing of Mo atoms, as depicted in Figure 3b3, which reveals the hexagonal structural arrangement of Mo atoms when viewed along the [001] direction. In these images, C atoms exhibit negligible contrast, making only the Mo atoms visible. In our STEM images, the lattice contrast appears less defined, primarily due to the coexistence of Mo_1.33_C layers with residual ErF_3_, which diminishes the visibility of vacancy ordering. To understand vacancy formation in Mo_1.33_C@Er, we compared the simulated [001] projections of the (Mo_2/3_Er_1/3_)_2_AlC phase (Figure S6a) and the corresponding Mo_1.33_C model (Figure S6b), generated using VESTA, with the experimental HRSTEM image of the prepared sample (Figure 3b3). In the (Mo_2/3_Er_1/3_)_2_AlC structure, Er atoms occupy the centers of the Mo hexagons (see Figure S6a). During etching, these Er atoms are removed, creating characteristic vacancies at the hexagon centers in the i-MXene model (Figure S6b). When we compare this model with the experimental HRSTEM image of Mo_1.33_C@Er (Figure 3b3), we observe the expected hexagonal arrangement of Mo atoms with empty centers, indicating the removal of Er and the formation of vacancies. STEM HAADF EDS mapping reveals that bright regions (in Figure 3c) correspond to Er and F, as shown in the mapping data (Figure 3c2,c4), while the lighter areas correspond to Mo (Figure 3c1), as seen from the Mo mapping data. The Er and F mappings appear identical, whereas Figure 3c3 shows that Mo and Er atoms are unevenly distributed. This suggests that Er atoms were etched out from the in-plane-ordered (Mo_2/3_Er_1/3_)_2_AlC structure but persisted in the final sample as ErF_3_, since this byproduct is insoluble in the washing solvent. As a result, the product is a mixture of Mo_1.33_C and ErF_3_. Additionally, O and C mapping signals were detected (Figure 3c5,c6), originating from surface functional groups and the overall carbon framework of Mo_1.33_C@Er.

**Figure 3:**
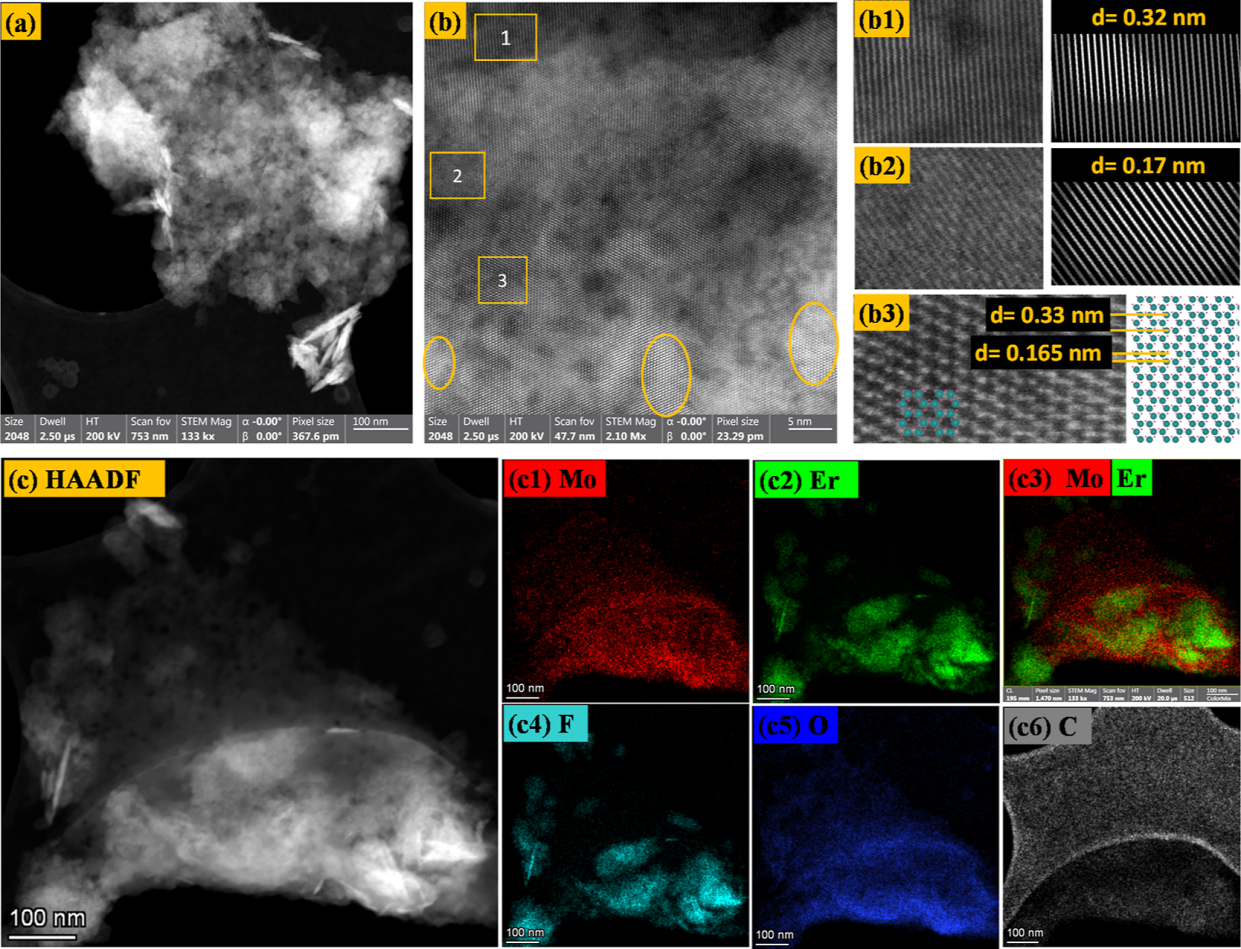
STEM HAADF micrograph images of Mo_1.33_C@Er at (a) 100 nm and (b) 5 nm resolution, (b1–b3) enlarged view of small areas 1, 2, and 3 marked in image (b). (c) STEM HAADF image at 100 nm with its EDS mapping (c1) Mo, (c2) Er, (c3) Mo, Er, (c4) F, (c5) O, and (c6) C of the prepared sample.

### 6.2 Electrochemical Performance of Mo_1.33_C@Er 2D i-MXene

To get insight into the electrochemical behavior of prepared Mo_1.33_C@Er, CV, GCD, and EIS were performed in a three-electrode system using 1 M H_2_SO_4_ electrolyte. In Figure 4a, Mo_1.33_C@Er exhibited a significant increase in current density compared to its precursor, (Mo_2/3_Er_1/3_)_2_AlC. Both Mo_1.33_C@Er and (Mo_2/3_Er_1/3_)_2_AlC electrodes were swept over a potential range of 0.0–0.4 V, where the current response and enclosed CV curve area directly correlate with their energy storage capability. The enhanced current response in Mo_1.33_C@Er suggests that the removal of aluminum during the etching process resulted in a more open and conductive surface. Using the formula $C=\frac{\int_{V_{1}}^{V_{2}}Idv}{2sm\Delta V}$ , where *C* is the specific capacitance, $\int_{V_{1}}^{V_{2}}Idv$ is the integral CV curve area, *s* is the scan rate, *m* is the mass of the active material, and Δ*V* is the potential window,^60,61^ the (Mo_2/3_Er_1/3_)_2_AlC i-MAX phase showed a specific capacitance of 78.8 mF/g, while Mo_1.33_C@Er exhibited a significantly higher value of 1904 mF/g, marking a 24-fold improvement. When the potential window was extended to 0.5 V, the specific capacitance of Mo_1.33_C@Er further increased to 2214.8 mF/g. The same could not be performed with (Mo_2/3_Er_1/3_)_2_AlC because when scanned over a wider potential window, it showed instability in CV behavior (Figure S7). Starting from 0.0 to 0.5 V, the charging and discharging curves displayed an unusual overlap or crossing, indicating poor electron kinetics and low conductivity. However, Mo_1.33_C@Er maintained stable CV profiles up to 0.0–0.7 V, demonstrating better charge storage performance across an extended applied potential range (Figure S8). The significant increase in capacitance upon converting i-MAX to i-MXene could be explained by the emergence of termination-derived surface electronic states, as observed in the DFT analysis, which then promotes rapid faradaic charge storage. Mo_1.33_C@Er exhibits Mo 4d/O 2p hybridization at the Fermi level, creating high-density surface electronic states that act as fast redox-active sites. In Figure 4b, the CV curves of Mo_1.33_C@Er were recorded at increasing scan rates of within 2 mV/s to 120 mV/s. The typical increase in the CV area and decline in specific capacitance with increasing scan rate were observed. The calculated values were 22.1, 20.2, 11.7, 8.4, 6.8, 5.7, 4.9, and 4.4 F/g at scan rates of 2, 5, 20, 40, 60, 80, 100, and 120 mV/s, respectively. The GCD profiles of Mo_1.33_C@Er (Figure 4c) exhibit stable charge–discharge behavior with good Coulombic efficiency, delivering specific capacitances of 35.6, 29.9, 13.8, 23.6, and 15.8 F g^–1^ at current densities of 2, 3, 4, 5, and 10 A g^–1^, respectively. Under continuous cycling, the material preserves 86.7% of its initial capacitance after 1000 cycles, which gradually declines to 62.4% after 5000 cycles (Figure S9). Considering the heterogeneous surface chemistry of Mo_1.33_C@Er as shown in the Raman spectroscopy, XPS, and DFT results, the presence of irreversible surface redox processes could be likely, which manifested in the early cycling stages. As these reactions become exhausted or inactive over time, the available active sites decrease, resulting in the progressive loss of capacitance. The relationship between the scan rate and specific capacitance was studied to determine the charge storage mechanisms in Mo_1.33_C@Er. The Trasatti method was used to distinguish between electric double layer capacitance (EDLC) and pseudocapacitance (PS). This technique takes advantage of the different dependencies of EDLC and PS on the scan rate due to their distinct charge storage mechanisms.^62,63^ At higher scan rates (*s*), electrolyte ions are restricted to interacting only with the outer surface of the electrode material, making capacitance (*C*) primarily dependent on surface interactions between the outer electrode and the electrolyte.^62^ Consequently, the *y*-intercept of the *s* ^–1/2^ vs *C* plot represents the capacitance contribution from surface-controlled EDLC (Figure S10a). This dominance could again be traced to the surface-localized electronic states of the Mo 4d/O 2p hybridization at the Fermi level, as mentioned in the electronic properties. Conversely, at lower scan rates, diffusion effects take precedence, allowing electrolyte ions to reach both the inner and outer electrode surfaces.^62^ In this scenario, the *y*-intercept of the linear fit from *s* ^1/2^ vs 1/*C* corresponds to the total capacitance (Figure S10b). The difference between the total capacitance and the contribution from EDLC accounts for the pseudocapacitive contribution. Based on these calculations, pseudocapacitance dominates the storage mechanism, constituting 98.05% of the total capacitance, while the remaining 1.95% is attributed to EDLC (Figure S10c). To further understand the charge storage mechanism and kinetics of Mo_1.33_C@Er, EIS was conducted, and the corresponding Nyquist plot is shown in Figure 4d. The fitted equivalent circuit, *R*_1_ + (*C*_2_/*R*_2_) + (*C*_3_/*R*_3_) + *W*_4_, using the Zfit and PseudoC functions of Biologic EC-Lab software provided valuable information on the charge transfer, pseudocapacitive behavior, and ion diffusion limitations of Mo_1.33_C@Er.^64,65^ The fitted parameters are summarized in Table 1. The low *R*_1_ value (0.8965 Ω), which is the solution resistance, suggests minimal resistance from the ionic environment. The spectrum exhibits a large semicircle in the high-to-medium frequency range, indicative of high charge-transfer resistance (*R*_2_ = 152.1 Ω, *R*_3_ = 288.1 Ω). This suggests that while redox reactions occur, electron transport within the material is hindered, likely due to the presence of ErF_3_ that could act as an insulating layer.^66^ Combining the insight gained from EIS and Trasatti analysis, it could be suggested that Mo_1.33_C@Er displays distinctly surface-dominated pseudocapacitive behavior.

**Figure 4:**
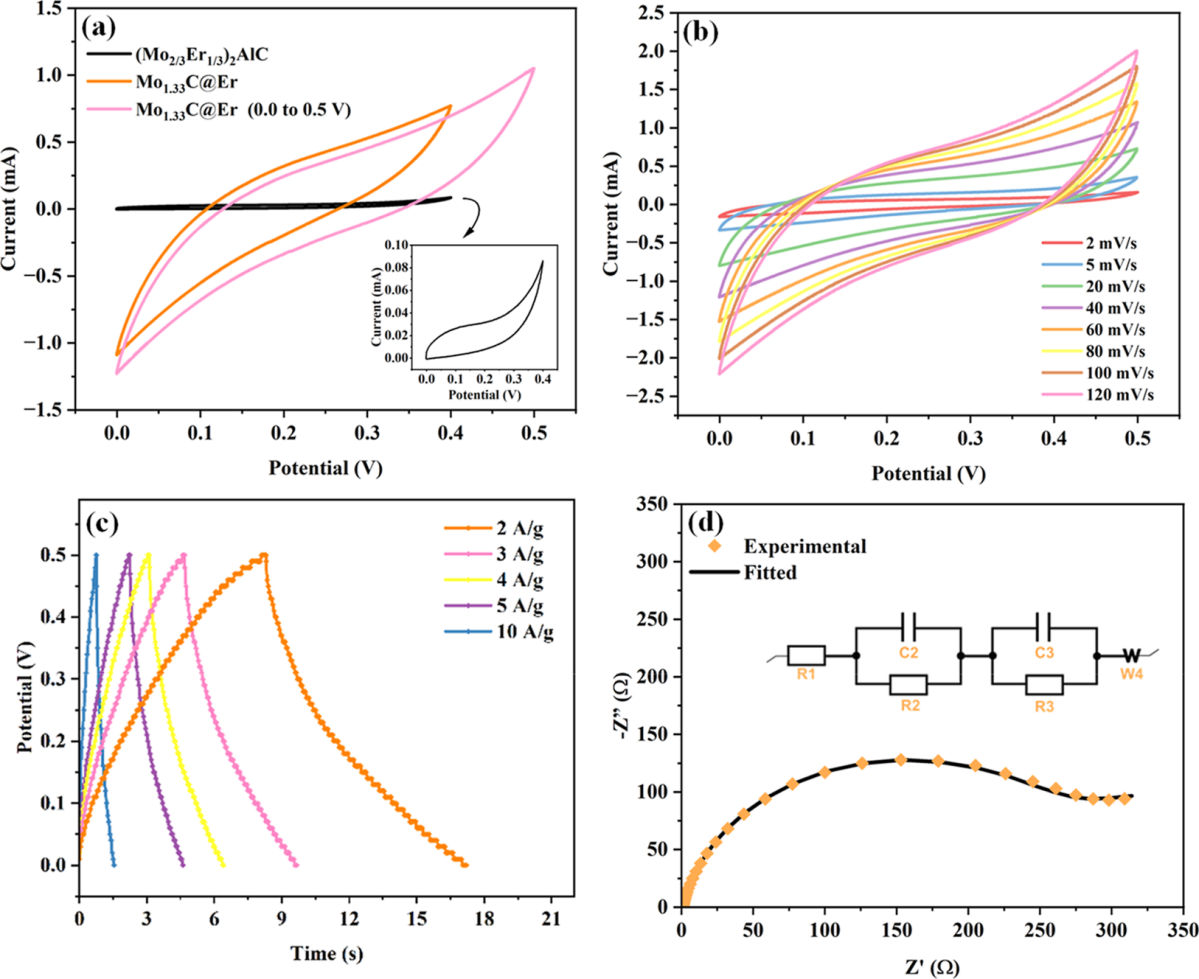
(a) Comparative cyclic voltammetry (CV) curves of the (Mo_2/3_Er_1/3_)_2_AlC i-MAX phase and Mo_1.33_C@Er at a scan rate of 100 mV/s, (b) CV curves of Mo_1.33_C@Er at increasing scan rates, (c) galvanostatic charge and discharge (GCD) curves of Mo_1.33_C@Er at increasing current density, and (d) Nyquist plot of the electrochemical impedance of Mo_1.33_C@Er, fitted with an equivalent circuit.

**Table 1:** Equivalent Circuit Fitting Parameters from the EIS Measurement of Mo_1.33_C@Er

*R*_1_ (Ω)	*C*_2_ (F)	*R*_2_ (Ω)	*C*_3_ (F)	*R*_3_ (Ω)	*W*_4_ (Ω s^–1/2^)
0.8965	1.914 × 10^–3^	152.1	50.9 × 10^–6^	288.1	0.3343 × 10^–12^

### 6.3 Electronic Properties of Mo_1.33_C 2D i-MXene

Recognizing the impact of surface terminations on MXene electronic properties, we investigated Mo_1.33_C through DFT calculations, considering both pristine and functionalized forms. The optimized structures remain monoclinic with C_2_/c crystal symmetry. The eV/atom normalized formation energy is the largest negative (−0.3491) for oxygen–fluorine mix-terminated Mo_1.33_C­(OF)*x* among the bare, –O, –F, and –O/–F mix-terminated i-MXenes considered in this study, indicating that oxygen–fluorine mix-terminated i-MXene is energetically favorable. To measure how strongly the functional groups are bonded with the parent i-MXene, the binding energy *E*_B_ per functional group (T_*x*_) is calculated using the following equation:

$$E_{B/T_{x}}=\frac{1}{n}\left[E\left({Mo}_{1.33}CT_{x}\right)-E\left({Mo}_{1.33}C\right)-nE\left(T_{x}\right)\right]$$

where *E*() denotes the energy of respective systems in their stable states. With $E_{B/T_{x}}=-1.071\;eV/T_{x}$ , –O/–F mix-terminated i-MXene has the largest negative value for $E_{B/T_{x}}$ . The $E_{B/T_{x}}$ follows the *E*_B/OF_ < *E*_B/F_ < *E*_B/O_ inequality, indicating that fluorine bonds more strongly than oxygen, and the bonding is even stronger in the mix-terminated one. Figure 5a–d represents band structures, and Figure 5e–h shows orbital projected density of states for bare, O-terminated, F-terminated, and –O and –F mix-terminated Mo_1.33_C. The energy level is shifted by the Fermi level (*E*_F_). The chosen *k*-point mesh follows the path of Γ (0,0,0) → *Z* (0,–1/2,1/2) → *M* (−1/2,–1/2,1/2) → *A* (−1/2,0,0) → Γ (0,0,0) → *V* (0,0,1/2). Band structures show that bare and all functionalized Mo_1.33_C are metallic. Nonzero density of states at the Fermi level confirms the metallic nature. At the Fermi level and conduction band, the density states are dominated by Mo 4d states for each of the structures. At the valence band, we witness the hybridization of Mo 4d states with C 2p states, O 2p states, and/or F 2p states. O 2p’s hybridization is stronger in O-terminated Mo_1.33_C than F 2p’s hybridization in F-terminated Mo_1.33_C. In O-terminated Mo_1.33_C, O 2p has a significant contribution at the Fermi level, while that contribution shifts to a higher energy value, making it significant in the conduction band. The metallic nature is also preserved in the –O/–F mix-terminated Mo_1.33_C. The mix-termination caused enhanced O 2p contribution in the conduction band compared to the other single-atom-type surface termination.

**Figure 5:**
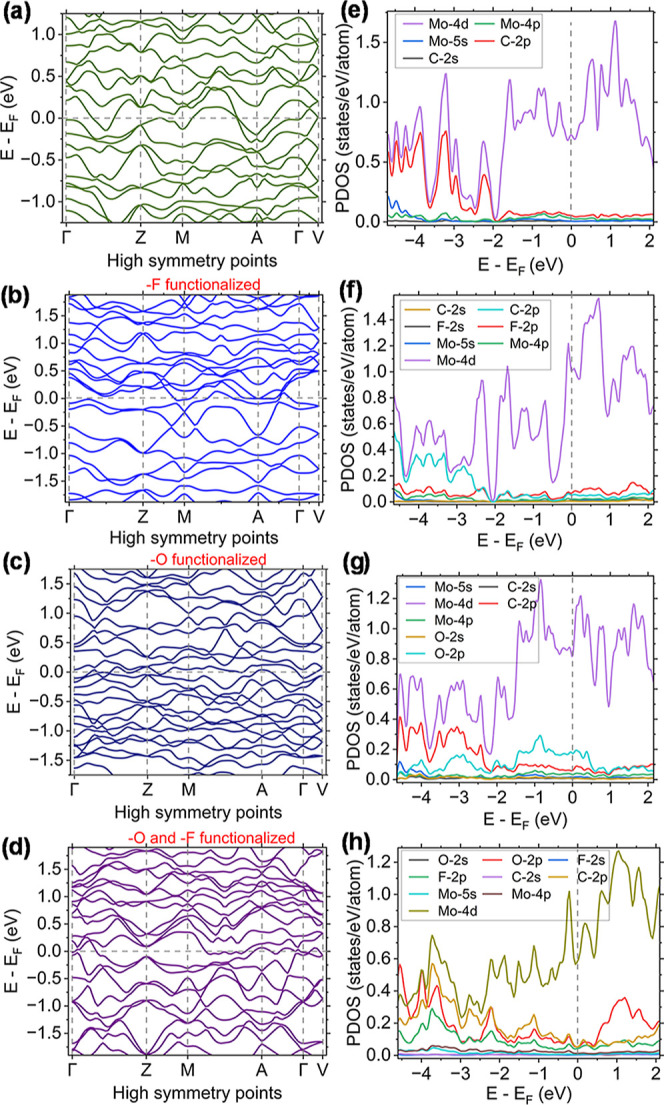
(a–d) Band structures and (e–h) orbital projected density of states for bare Mo_1.33_C, oxygen-functionalized Mo_1.33_C, fluorine-functionalized Mo_1.33_C, and mix-terminated Mo_1.33_C functionalized with oxygen and fluorine.

## 7 Conclusions

Using the LiF/HCl mild etching method, 2D i-MXene can be prepared; however, achieving a pure i-MXene from the (Mo_2/3_Er_1/3_)_2_AlC i-MAX phase presents significant challenges. This is primarily due to the formation of RE fluoride compounds (e.g., ErF_3_) in the fluorine-rich etching environment, driven by the strong electronegativity difference between the Er and F atoms. These fluoride byproducts are largely insoluble in HCl and deionized water, making them difficult to remove through conventional washing procedures. Results conclude that ErF_3_ remains embedded in Mo_1.33_C@Er due to the insolubility of this compound in common acids, emphasizing the need to refine washing procedures or synthesis routes for 2D i-MXenes derived from RE-based i-MAX phases. To achieve high-purity 2D i-MXenes, it is essential to evaluate the solubility of the resulting byproducts and, going forward, to assess the viability of different i-MAX precursors through comprehensive structural characterization. Additionally, Mo_1.33_C@Er exhibits a specific capacitance of 2.21 F/g at a scan rate of 2 mV/s and a potential window of 0–0.5 V. However, EIS measurements reveal a relatively high charge-transfer resistance, suggesting that electron transport is hindered by the presence of residual ErF_3_ byproducts, likely to form an insulating layer, thereby fading the overall electrochemical performance. Prior reports on Mo_1.33_C 2D i-MXene prepared from the (Mo_2/3_Sc_1/3_)_2_AlC i-MAX phase demonstrate that ordered vacancies can enable high capacitance and efficient ion transport.^21^ Therefore, we have framed the material as a promising candidate for electrochemical energy storage applications once the synthesis procedure is further improved. DFT calculations show both the bare and functionalized Mo_1.33_C are metallic. 4d states of Mo, 2p states of C, and 2p states of O and/or F have a significant contribution to the total density of states of Mo_1.33_CT_*x*_.

## Supplementary Material



## Notes

Raman lines for (Mo_2/3_Er_1/3_)_2_AlC i-MAX and Mo_1.33_C@Er i-MXene; electrochemical performance; SEM images; the XPS survey; O 1s, F 1s, and Cl 2p spectra; bluish-colored colloidal dispersion of delaminated Mo_1.33_C@Er i-MXene; STEM-HAADF image; EDS elemental maps; VESTA-generated structural models; cyclic voltammetry curves; cycling stability performance; GCD curves for the first and 5000th cycles; and EDLC and pseudocapacitive contributions of Mo_1.33_C@Er i-MXene (PDF)
