# Supplementary material for: 2D in-Plane Ordered MXene Nanosheets Derived from (Mo2/3Er1/3)2AlC Rare-Earth i‑MAX for Energy Storage Applications
Source: ACS Appl Nano Mater. 2026 Jan 6;9(2):1089–98. doi: 10.1021/acsanm.5c04789 (PMC12813977; doi:10.1021/acsanm.5c04789)
Supplement: Supplementary file 1 [file an5c04789_si_001.pdf]

2D in-Plane Ordered MXene Nanosheets Derived from $(Mo_{2/3}Er_{1/3})_2AlC$ Rare-Earth i-MAX for Energy Storage Applications

Nisha Hiralal Makani[1], Chandra M. Adhikari[1], Shanna Marie M. Alonzo[2], Bishnu Prasad Bastakoti[2], Binod K. Rai[3], Bhoj Raj Gautam[1*]

[1]*Department of Chemistry, Physics and Materials Science, Fayetteville State University, Fayetteville, North Carolina 28301, United States of America*

[2]*Department of Chemistry, North Carolina A&T State University, Greensboro, NC 27411, United States of America*

[3]*Savannah River National Laboratory, Aiken, South Carolina 29808, United States of America*

[*]*Email: bgautam@uncfsu.edu*

Table S1: Experimentally observed Raman lines for $(Mo_{2/3}Er_{1/3})_2AlC$ i-MAX and $Mo_{1.33}C$@Er i-MXene

$(Mo_{2/3}Er_{1/3})_2AlC$ (cm^{-1})	$Mo_{1.33}C$@Er (cm^{-1})
123.08 (Er)[1]	120.32 (Er) [1]
168.60 (Er-Mo-Al) [1]	154.12 (Er-Mo) [1]
180.40 (Mo) [1]	243.49 (Er-F) [2]
236.23 (Mo-Al) [1]	286.29 (Er-F) [2]
267.02 (Mo-Al) [1]	337.61 (Er-O) [3]
292.05 (Mo-Al) [1]	524.11 (Er-F)[2]
341.87 (Er-O) [3]	563.66 (C)
384.67 (Er-O) [3]	600.46(Mo-C)[4]
507.83 (C) [1]	669.55(Mo-O)[5]
560.90 (C)[1]	701.84 (Mo-C)[4]
598.95 (C)[1]	820.75 (Mo-O) [5]

610.72 (Mo-C) [4]	996.98 (Mo-O) [5]
826.50 (Mo-O) [5]	

Table S2: Comparison of the electrochemical performance of $Mo_{1.33}C$@Er i-MXene with literature results

i-MXenes	Electrolyte	Tested condition	Specific capacitance	References
$Mo_{1.33}Y_XC$	1M H_2SO_4	2 mV/s	120 F/g	[6]
$Mo_{1.33}C$@Sc	1M H_2SO_4	2 mV/s	330 F/g	[7]
$Mo_{1.33}C$@Y	1M H_2SO_4	1 A/g	225 F/g	[8]
$Mo_{1.33}C$@Gd	3M H_2SO_4	1 A/g	177 F/g	[8]
$Mo_{1.33}C$@Tb	3M H_2SO_4	1 A/g	189 F/g	[9]
$Mo_{1.33}C$@Dy	3M H_2SO_4	1 A/g	197 F/g	[8]
$Mo_{1.33}C$@Ho	3M H_2SO_4	1 A/g	228 F/g	[8]
$Mo_{1.33}C$@Er	3M H_2SO_4	1 A/g	171 F/g	[8]
$Mo_{1.33}C$@Er	1M H_2SO_4	2 A/g	35.6 F/g	This Work

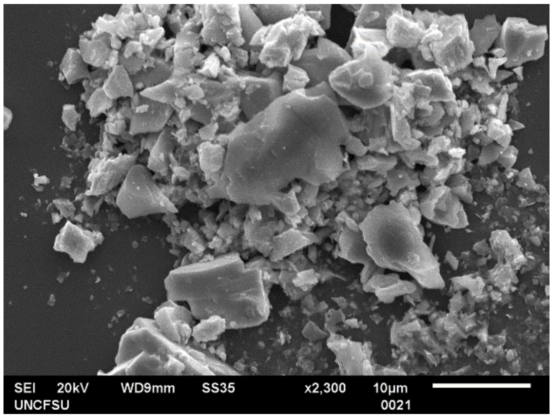

Figure S1: SEM image of $(Mo_{2/3}Er_{1/3})_2AlC$ i-MAX phase

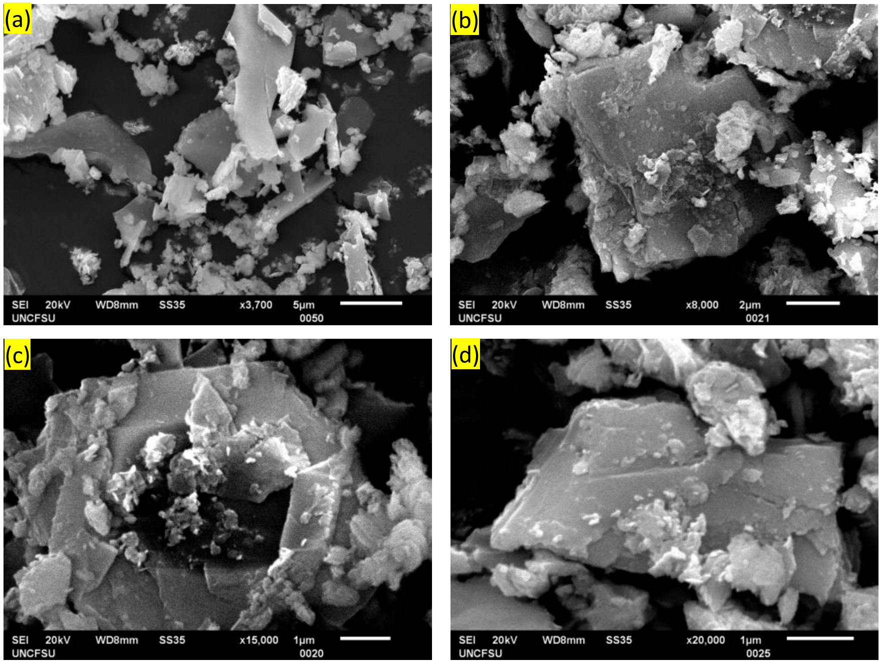

Figure S2: (a-d) SEM images of the 2D i-MXene captured from different regions and at varying magnifications

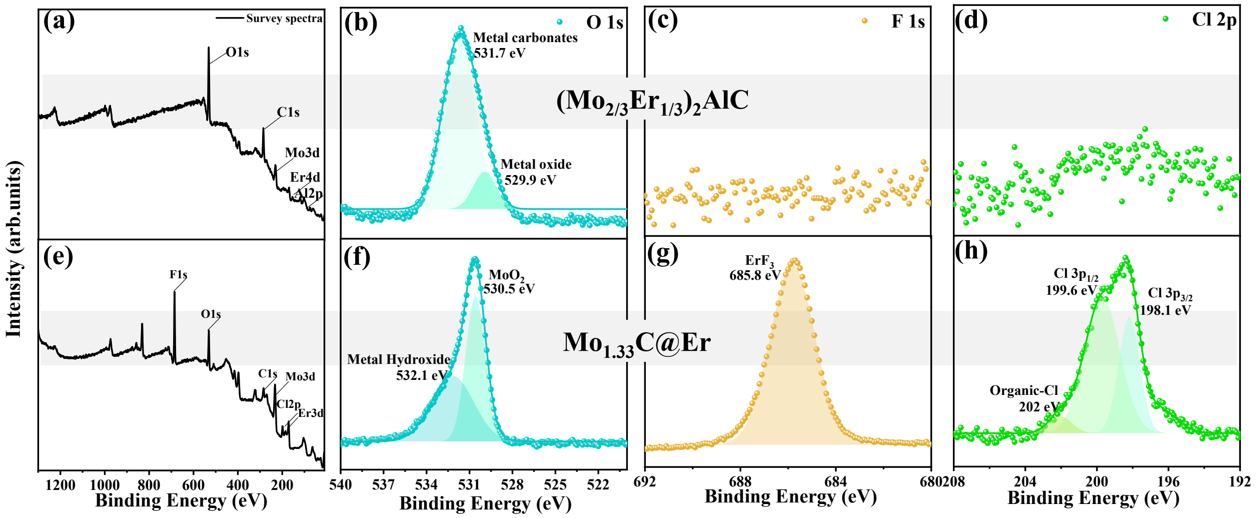

Figure S3: (a, e) Survey spectra, (b, f) O1s, (c, g) F1s, (d, h) Cl2p spectra of $(Mo_{2/3}Er_{1/3})_2AlC$ and $Mo_{1.33}C$@Er, respectively.

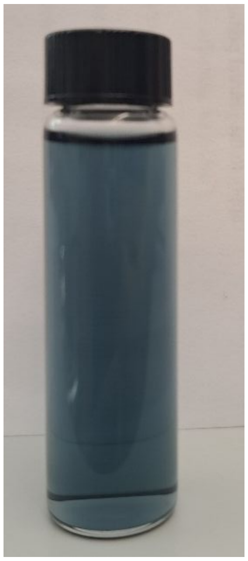

Figure S4: Bluish colored solution of delaminated $Mo_{1.33}C$@Er i-MXene using TBAOH.

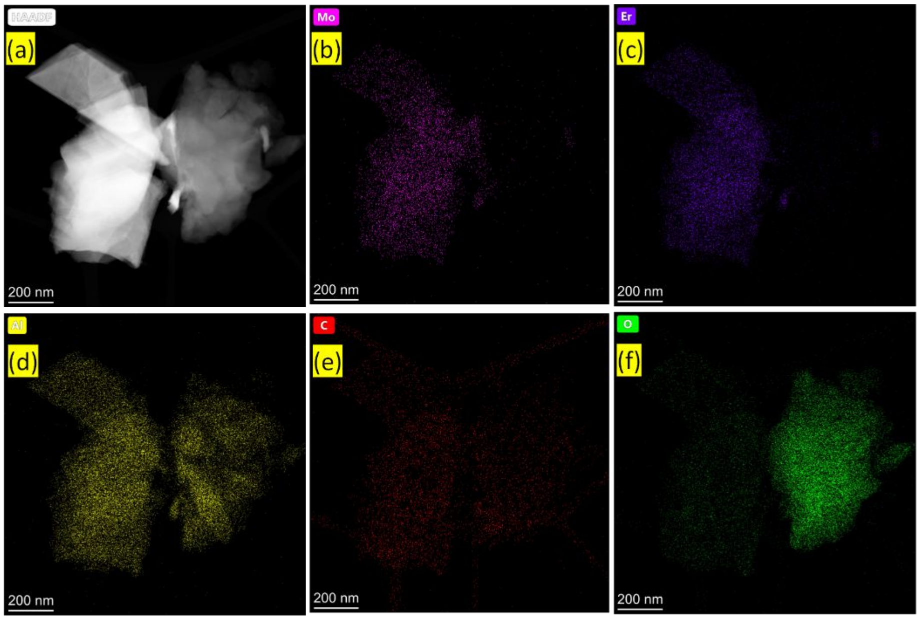

Figure S5: (a) STEM HAADF image at 200nm with its EDS mapping (b) Mo, (c) Er, (d) F, (e) C, and (f) O of $(Mo_{2/3}Er_{1/3})_2AlC$ i-MAX phase.

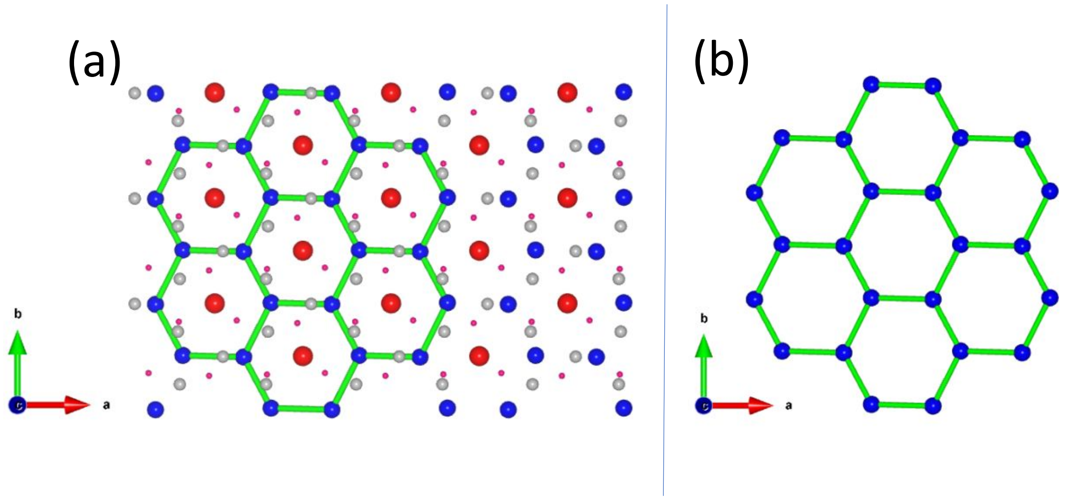

Figure S6: (a) [001] view of the i-MAX phase, (red atoms (Er), Blue atoms (Mo), grey atoms (Al), and pink atoms (C)), (b) corresponding i-MXene structure after Al and Er removal, (Blue atoms (Mo).

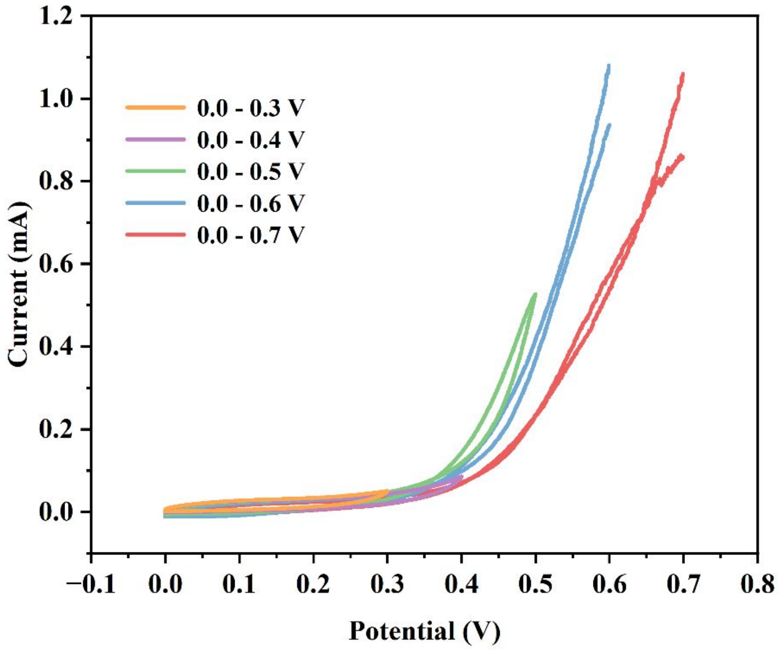

Figure S7: Cyclic voltammetry (CV) curves of $(Mo_{2/3}Er_{1/3})_2AlC$ i-MAX phase at various potential windows.

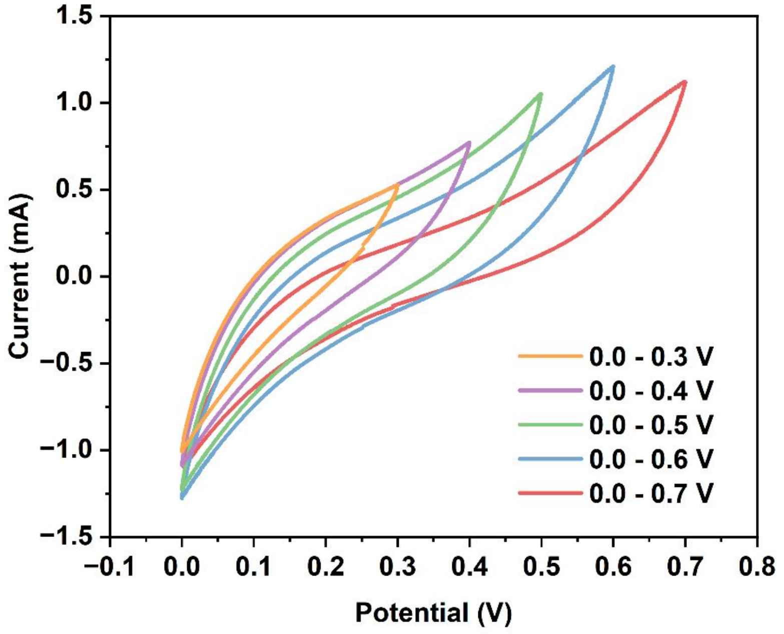

Figure S8: Cyclic voltammetry (CV) curves of $Mo_{1.33}C$@Er i-MXene at various potential windows.

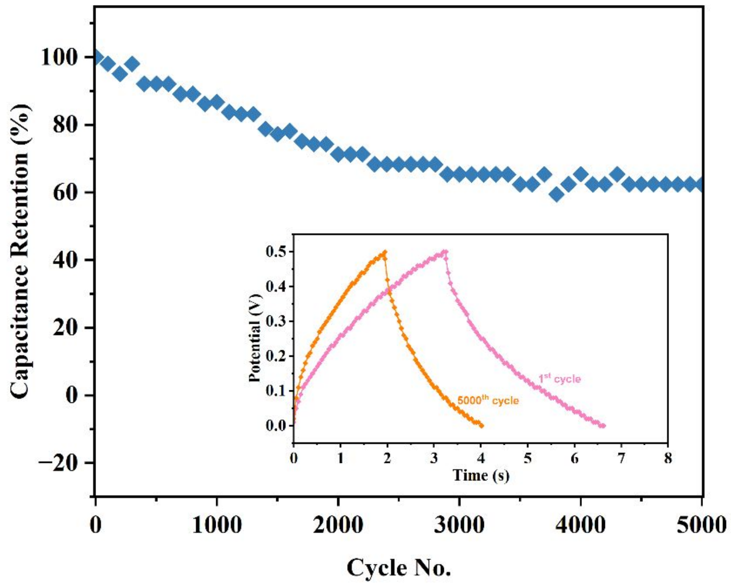

Figure S9: Cycling stability of $Mo_{1.33}C$@Er i-MXene at 3 A/g. Inset: GCD curves at the 1st and 5000th cycles.

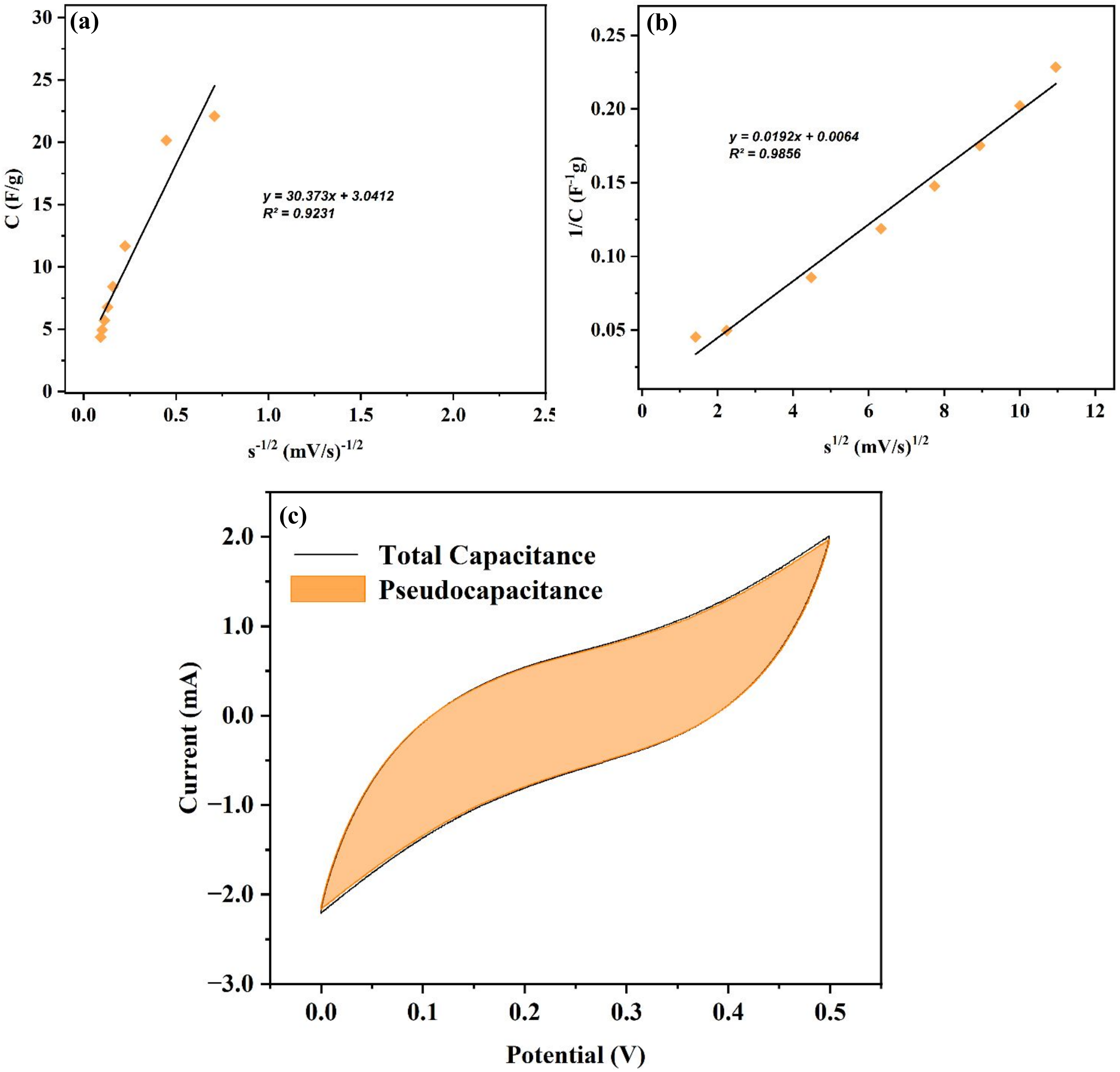

Figure S10: Capacitance contributions of EDLC and pseudocapacitance in $Mo_{1.33}C$@Er i-MXene using the Trasatti method. From the linear fits of (a) $s^{-1/2}$ vs. C and (b) $s^{1/2}$ vs. $1/C$, the percent contribution from EDLC and pseudocapacitance was determined to be 1.95% and 98.05%, respectively. (c) CV curve at 120 mV/s, emphasizing the predominant pseudocapacitive contribution.

References:

(1) Champagne, A.; Chaix-Pluchery, O.; Ouisse, T.; Pinek, D.; Gélard, I.; Jouffret, L.; Barbier, M.; Wilhelm, F.; Tao, Q.; Lu, J.; Rosen, J.; Barsoum, M. W.; Charlier, J. C. First-Order Raman Scattering of Rare-Earth Containing i -MAX Single Crystals (Mo2/3RE1/3)2AlC (RE=Nd, Gd, Dy, Ho, Er). *Phys. Rev. Mater.* **2019**, *3* (5), 053609.

(2) Rotereau, K.; Daniel, P.; Gesland, J. Y. Vibrational and Electronic Properties of the Lanthanide Trifluorides GdF3, TbF3, ErF3 and YbF3 Studied by Raman Spectroscopy. *J. Phys. Chem. Solids* **1998**, *59* (6–7), 969–980.

(3) Tomar, R.; Kumar, P.; Kumar, A.; Kumar, A.; Kumar, P.; Pant, R. P.; Asokan, K. Investigations on Structural and Magnetic Properties of Mn Doped Er2O3. *Solid State Sci.* **2017**, *67*, 8–12.

(4) Li, T.; Luo, W.; Kitadai, H.; Wang, X.; Ling, X. Probing the Domain Architecture in 2D α-Mo 2 C via Polarized Raman Spectroscopy. *Adv. Mater.* **2019**, *31* (8), 1807160.

(5) Reddy, R. K. K.; Kailasa, S.; Rani, B. G.; Jayarambabu, N.; Yasuhiko, H.; Ramana, G. V.; Rao, K. V. Hydrothermal Approached 1-D Molybdenum Oxide Nanostructures for High-Performance Supercapacitor Application. *SN Appl. Sci.* **2019**, *1* (11), 1–9.

(6) Persson, I.; el Ghazaly, A.; Tao, Q.; Halim, J.; Kota, S.; Darakchieva, V.; Palisaitis, J.; Barsoum, M. W.; Rosen, J.; Å Persson, P. O.; Persson, I.; el Ghazaly, A.; Tao, Q.; Halim, J.; Palisaitis, J.; Rosen, J.; Å Persson, P. O.; Kota, S.; Barsoum, M. W.; Darakchieva, V. Tailoring Structure, Composition, and Energy Storage Properties of MXenes from Selective Etching of In-Plane, Chemically Ordered MAX Phases. *Small* **2018**, *14* (17), 1703676.

(7) Tao, Q.; Dahlqvist, M.; Lu, J.; Kota, S.; Meshkian, R.; Halim, J.; Palisaitis, J.; Hultman, L.; Barsoum, M. W.; Persson, P. O. Å.; Rosen, J. Two-Dimensional Mo1.33C MXene with Divacancy Ordering Prepared from Parent 3D Laminate with in-Plane Chemical Ordering. *Nat. Commun.* **2017**, *8* (1), 1–7.

(8) Yang, J.; Yao, G.; Sun, S.; Chen, Z.; Yuan, S.; Wu, K.; Fu, X.; Wang, Q.; Cui, W. Structural, Magnetic Properties of in-Plane Chemically Ordered (Mo2/3R1/3)2AlC (R = Gd, Tb, Dy, Ho, Er and Y) MAX Phase and Enhanced Capacitance of Mo1.33C MXene Derivatives. *Carbon* **2021**, *179*, 104–110.

(9) Yang, J.; Liu, R.; Jia, N.; Wu, K.; Fu, X.; Wang, Q.; Cui, W. Novel W-Based in-Plane Chemically Ordered (W2/3R1/3)2AlC (R = Gd, Tb, Dy, Ho, Er, Tm and Lu) MAX Phases and Their 2D W1.33C MXene Derivatives. *Carbon* **2021**, *183*, 76–83.
